# Deep Learning-Based Automatic Detection of Ships: An Experimental Study Using Satellite Images

**DOI:** 10.3390/jimaging8070182

**Published:** 2022-06-28

**Authors:** Krishna Patel, Chintan Bhatt, Pier Luigi Mazzeo

**Affiliations:** 1 Department of Computer Science & Engineering, Devang Patel Institute of Advance Technology and Research (DEPSTAR), CHARUSAT Campus, Charotar University of Science and Technology (CHARUSAT), Changa 388421, India; krishnapatel.ce@charusat.ac.in; 2 U & P U. Patel Department of Computer Engineering, Chandubhai S Patel Institute of Technology (CSPIT), CHARUSAT Campus, Charotar University of Science and Technology (CHARUSAT), Changa 388421, India; 3 Institute of Applied Sciences and Intelligent Systems, National Research Council of Italy, 73100 Lecce, Italy

**Keywords:** image classification, deep learning, remote sensing, convolutional neural networks, ships detection, satellite images, surveillance

## Abstract

The remote sensing surveillance of maritime areas represents an essential task for both security and environmental reasons. Recently, learning strategies belonging to the field of machine learning (ML) have become a niche of interest for the community of remote sensing. Specifically, a major challenge is the automatic classification of ships from satellite imagery, which is needed for traffic surveillance systems, the protection of illegal fisheries, control systems of oil discharge, and the monitoring of sea pollution. Deep learning (DL) is a branch of ML that has emerged in the last few years as a result of advancements in digital technology and data availability. DL has shown capacity and efficacy in tackling difficult learning tasks that were previously intractable. Specifically, DL methods, such as convolutional neural networks (CNNs), have been reported to be efficient in image detection and recognition applications. In this paper, we focused on the development of an automatic ship detection (ASD) approach by using DL methods for assessing the Airbus ship dataset (composed of about 40 K satellite images). The paper explores and analyzes the distinct variations of the YOLO algorithm for the detection of ships from satellite images. A comparison of different versions of YOLO algorithms for ship detection, such as YOLOv3, YOLOv4, and YOLOv5, is presented, after training them on a personal computer with a large dataset of satellite images of the Airbus Ship Challenge and Shipsnet. The differences between the algorithms could be observed on the personal computer. We have confirmed that these algorithms can be used for effective ship detection from satellite images. The conclusion drawn from the conducted research is that the YOLOv5 object detection algorithm outperforms the other versions of the YOLO algorithm, i.e., YOLOv4 and YOLOv3 in terms accuracy of 99% for YOLOv5 compared to 98% and 97% respectively for YOLOv4 and YOLOv3.

## 1. Introduction

In the last years, machine learning (ML) has become widespread and many artificial intelligence (AI) applications, have an impact on our daily life. These novel developments in AI are supported by a change in the approach to algorithm design. Specifically, data-driven ML has required a considerable level of manual feature engineering that utilized specialist knowledge from specific domains. The investigation of these data-driven techniques is known as deep learning (DL) [1].

In terms of representation learning, DL is an excellent approach to be considered in the object detection use cases. Therefore, DL adopts more advanced schemes, such as transfer learning (TL) [2], which refers to using a model which had been trained for a certain task to perform another task.

The community of remote sensing (RS) is increasingly interested in addressing the challenges of image detection through the application of ML and DL techniques. Ship detection is of crucial importance to maritime surveillance, and can assist in monitoring and controlling illegal fishing, marine traffic, and similar activities along the sea boundary. In the last decades, automatic ship detection (ASD) has been a major challenge for researchers in the area of RS, and recent advances in RS technology have had a significant impact on the variety of its application areas, including safety, illegal trading, pollution, spills, and oil slick monitoring. [1,3,4]. Nevertheless, there are still many drawbacks to be addressed, to create accurate and robust automatic identification systems (AIS), capable of dealing with complex scenarios, that is, those with variability in the shape and size of the target object, e.g., ASD.

ASD utilizes both extremely high frequency (HF) and the Global Positioning System (GPS) to wirelessly identify the ship. However, not all ships are expected to have transponders, and some are switched off on purpose to prevent radar detection. Instead, synthetic aperture radar (SAR) may be used, which obtains RS imagery by using radar signals. Unlike visible light, radar signals are unaffected by variation in weather conditions, such as rain or clouds.

On the one hand, optical images are of a higher spatial resolution than most of the SAR images [5], making identification and recognition possible, as great improvements have been made in the radar field (COSMO-SkyMed spotlight reaches to 1 m resolution). However, in case the ASD or the Vessel Monitoring System (VMS) system are unable to classify the ship, it could suggest that the ship is potentially engaged in illegal activity or could be considered as a different shape of floating object. Images of spatial resolution, other than those derived with SAR, are now possible due to the recent advances in visible spectrum sensor technology. Several satellites (e.g., SPOT, RedEye, LandSat, QuickBird, and CBERS) are equipped with them. The main drawback of this type of images is that it is suggested not to be used at night or in low light conditions or when it is cloudy. In addition, SAR requires a greater number of pairs of antenna or scan positions, also suitable for slowly moving objects.

The use of SAR for ASD tasks has been extensively researched in the last two decades [6,7,8]. Unlike the variety of feasibility studies for ASD based on SAR, optical imagery has been used in few research studies. Optical sensor developments have helped us to partially overcome the disadvantages of SAR-based methods, and the authors focused on discovering the steps to be taken to gain improved classification accuracy, while also addressing the issues of real-time ASD [7]. Specifically, the detection of oriented objects from satellite photography is a complex task since the targeted objects can be visible from arbitrary directions and they are normally tightly packed. Recently, arbitrary-oriented object detection has received a lot of attention since they often appear in natural scenes, satellite photos, and RS images [9,10]. Additionally, other contributions on SotA made use of known features extracted from photographs, by using both computer vision and image processing techniques.

In the last few years, DL methods, such as convolutional neural networks (CNNs), have been shown to be effective in both image detection and recognition applications [10,11,12]. One or more convolutional layers are used in CNNs, which are normally accompanied by pooling layers and optional, completely connected layers, on top [11,12]. Moreover, Dropout and other regularization approaches have been used [13]. It has been proven that methods from the SotA of DL, e.g., YOLO9000, were unable to both detect and properly determine the total number of ships of small size by using satellite images [14]. A standard back-propagation method can be used to train these networks, but it needs a labeled dataset to measure the loss function gradient [15,16].

In this paper, we propose to take a step further in addressing discussed drawbacks of AISs, in particular of ASD. Our solution based on a well-known CNN architecture is able to define most distinguishing features for the given task if compared with hand-crafted features. The proposed framework makes use of a preprocessing step to extract image features, which are then classified by using a CNN-based classifier. Specifically, different CNNs (R-CNN, ResNet, Unet, YOLOv3, and YOLOv5) were considered for the automatic detection of small size ships by using satellite images [17]. In order to assess our system, we tested it on a publicly available ship dataset composed of about 40 K satellite images, also containing moving ships.

The main contribution of this work is: (i) computation of data from satellite imagery; and (ii) successful detection of ships using the traditional YOLOv5 algorithm and comparing obtained results between the YOLOv3 and YOLOv4 with up to date well known CNN architectures.

The structure of the paper is as follows. Section 2 provides a brief summary of the SotA on ASD methods based on satellite images, also including those methods that use DL strategies for classification. Subsequently, Section 3 introduces our proposal for ASD using DL. Section 4 provides both the considered datasets and a comprehensive analysis of the reported results derived from the conducted experiments. Finally, Section 5 provides the more relevant conclusions drawn from this study and outlines lines of research to be addressed in the future.

## 2. State-of-the-Art (SotA) on ASD

This section provides a brief overview of the SotA on ASD. Specifically, the contributions from the DL field have been of special interest to us for the purposes of this research.

Due to natural variability in the ocean waves and the weather (e.g., clouds and rain), automatic ship detection and recognition represent a complex task if addressed with common approaches, e.g., those from computer vision or image processing. In particular, complex detection scenarios are those with variability in the shape and size of the target object, together with the behavior of natural resources, such as islets, cliffs, sandbanks, and coral reefs.

Especially difficult conditions, such as variability in day lighting or atmospheric conditions, as well as images that include land area, pose an issue for conventional methods, as these images often produce false positives. As a result, many of the approaches have stopped making use of these types of source images in the datasets [18,19,20,21,22,23]. Other approaches use a geographical information mask to disregard land areas or to differentiate previously seen sea and land zones, but even in this scenario, tiny islets and islands are not identified and filtered out and may be mistaken in the recognition step [17]. Furthermore, this kind of approach is only applicable when using satellite images.

Regarding DL, there is no general code of practice for deciding the best choice of net-work architecture, the number of neurons per layer, and the total amount of layers more suitable to use to properly address a given classification problem [23]. Specifically, DL is aimed at making multilayered neural networks to model high-degree representations of input statistics. These statistics are given to the first layer in order to transform them and next transfer the result to the next layer, then repeating the procedure till the remaining layers produce the desired outcome [24]. The first transition layers provide low-level features (e.g., edges, corners, and gradients, among others) at the same time as the subsequent layers produce a growing high-degree representation of the maximum salient or consult-ant features. Then, DL has the benefit of no longer requiring hand-engineered characteristic extraction. As an alternative, the most crucial capabilities are found out from the raw input records, and this is frequently known as “representation learning” [25,26,27,28,29,30].

In [31,32,33], several methods for optical RS using DL have been recently contributed. These approaches were proposed to detect objects and classify scenes in optical satellite imagery such as residential zones, forest, and agricultural area. For instance, CNN activations from the final convolutional layer were created at a couple of scales, after which they were encoded into worldwide picture capabilities using widely used encoding methods to feed a classifier [34]. The approach was aimed at detecting ships in a harbor.

In [35], UNet model was used for the segmentation of ship from the SAR images. This paper proposed the UNet model and tested on the Airbus ship Challenge Dataset from Kaggle. The result showed the F2-score of approximately 0.82 on different IoU threshold values.

For the identification of ships from the Airbus satellite image dataset, Jaafar Alghazo [36] developed two CNN-based deep learning models, namely CNN model 1 and CNN model 2. The proposed methods could be utilised to address a variety of maritime-related issues, such as illegal fishing, maritime resource surveillance, and so on. The model had a maximum accuracy of 89%.

In [37], a new, deep learning-based, faster region-based convolutional neural network (Faster R-CNN) technique for detecting ships from two satellite image datasets, the 1786C-band Sentinel-1 and RADARSAT-2 vertical polarization, was introduced. The precision and recall of the model were 89.23% and 89.14%, respectively.

Xin lou [38] proposed a generative transfer learning method that combined knowledge transfer and ship detection. For ship detection, the images generated by the knowledge transfer module were fed into a CNN-based detector model. The SAR Ship Detection Dataset (SSDD) and AIR-SARShip-1.0 Dataset were used in the experiment.

**Figure 1:**
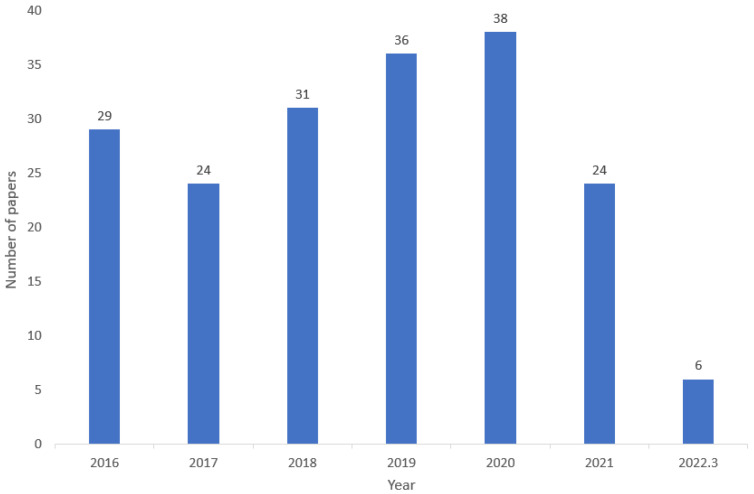
The number of papers on Ship Detection irrespective of region and country, (from 2016–2022 (March month)).

Figure 1 presents the total number of papers published for object detection over the past six years until this year. Unlike approximations, such as VGG-16/19, AlexNet, and VGG-F/M/S, which were applied to scene classification, our proposal is based on more suitable models, such as ResNet and YOLO, which have demonstrated to be more appropriate for ASD [34]. Table 1 demonstrates the comparative analysis of the work carried out on ship detection using the different datasets. Finally, our approach presents a methodology that employs CNN technology to extract various distinct features from satellite imagery, which are then classified as ships. The next section introduces the details of our ASD framework.

## 3. Methodology

This section provides the specific design of our ASD system using DL. In particular, it is explained how to identify scenes with or without ships using the proposed process. First, the description will go through the dataset used in the paper and the most up-to-date CNN algorithms (CNN, Region based CNN, ResNet. U-net). The various deep learning algorithms (YOLOv3, YOLOv4, and YOLOv5) are then introduced and evaluated.

### 3.1. Dataset

#### 3.1.1. Shipsnet Dataset

In this paper, the experiment is carried out on the ship images taken from a satellite images dataset which was collected by the Kaggle platform [42]. These images show the surface of earth, including roads, farmland, buildings, and other objects. PlanetScope has obtained these satellite images of the San Pedro Bay and San Francisco Bay located in the districts of California. It contains 4 k RGB images with a pixel resolution of 80 × 80 for two distinct classes: “ship” and “non-ship”.

Table 2 represents the total number of the images in each of the class (Ship and Non-ship). The images that contain a ship must show the entire appearance of a ship. It may be possible that ships are in a different orientation and have different sizes, while they may also have atmospheric noises.

Table 3 demonstrates each class-wise image. A non-ship class is made up of one or more of the following three elements: (1) bright pixels or strong linear features create a noise; (2) part of the ship in the image, and (3) other samples of various features, such as buildings, roads, water, and so on.

#### 3.1.2. Airbus Ship Dataset from Kaggle

**Figure 2:**
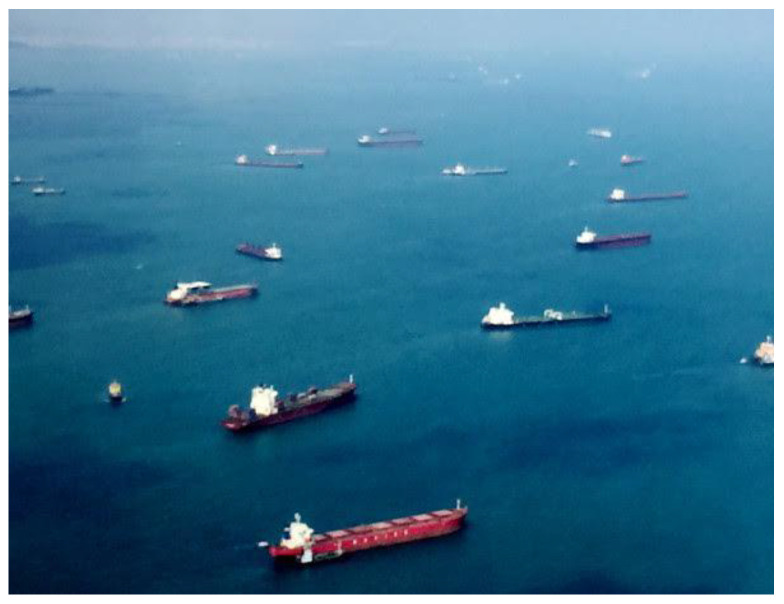
Image extracted from the dataset of ships.

**Figure 3:**
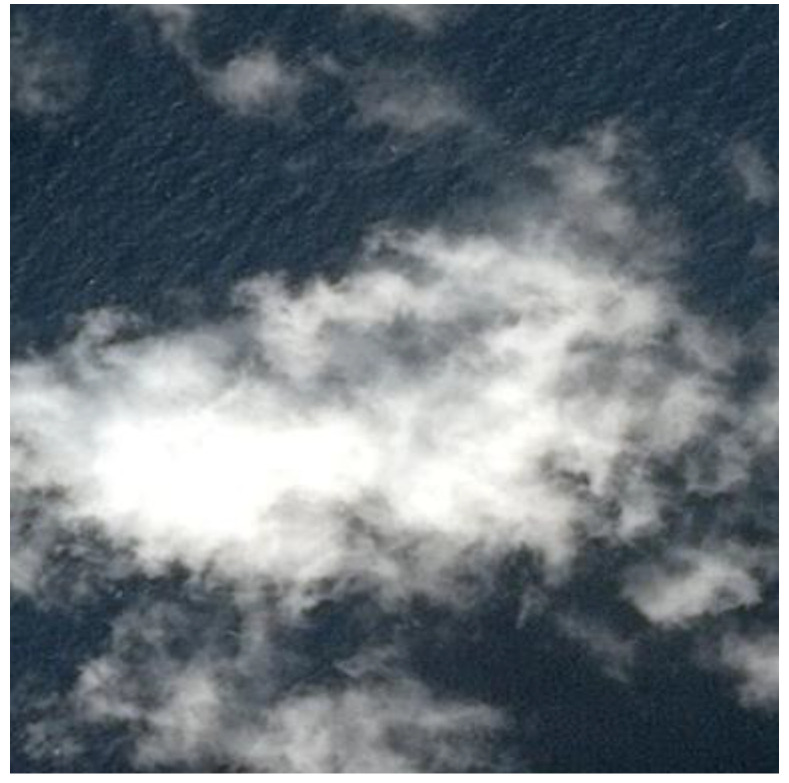
Dataset Image without ship.

The dataset used in this paper is from the Kaggle platform Airbus Ship Detection Challenge [43]. The dataset has RGB images with the resolution of 768 × 768 pixels. It also includes the encoded pixels in satellite photos that represent ship locations. The decoded pixels were transformed into binary masks, with 1 representing “ship” and 0 representing “no ship”. If a mask with value 1 exists, it is converted to a bounding box by computing the mask’s four corner coordinates. All photos were resized to 256 × 256 pixels in order to save computing power. The axes in the original data were inverted, hence the x and y coordinates were flipped as well. The figures below (Figure 2 and Figure 3) are an example of an image with ship and without ship. Because this dataset consists of a vast number of images, only satellite images with at least one ship were used, resulting in approximately 47,259 satellite images. However, due to the high processing time and memory consumption constraints, we could only manage up to 10 K dataset images, 7000 images as the training images, and 5000 images as the test images from the entire dataset, which were used to test and evaluate the model’s performance.

### 3.2. Convolutional Neural Network

The most representative DL model is CNN. VGG16 was the popular CNN architecture used for the object detection. Feature map is the name given to every layer of CNN. The CNN input layer’s feature map is a three-dimensional matrices of pixel frequencies for various color channels (e.g., RGB) [28]. Every single neuron in the preceding layer is linked to a limited number of neurons in the layer above it (receptive field). The pre-trained model is able to process any test image that is of the same size as the pre-trained sample. If different sizes are given, rescaling or cropping operations must be performed [28]. Additionally, some traditional drawbacks which come under the category of computer vision can be reformulated as a high-dimensional matrix data transform issue and can be addressed from multiple viewpoints, e.g., due to the huge learning power of deep CNNs [44].

### 3.3. Region-Based CNN (R-CNN)

Methods for object detection based on area proposals have had a great deal of success in natural scene photos in recent years. The architecture for detecting an object is divided into two categories. The first categories rely on compiling a list of possible objects containing candidate area proposals. The second step focuses on fine-tuning the bounding box coordinates and identifying the first-stage candidate region proposals into object groups or contexts [45,46,47,48,49]. The region-based CNN (R-CNN) is the most significant technique amongst all the region proposal-based methods. This is the first point where CNN models are used to produce a number of unique features for the detection of objects, which results in a major performance improvement over previous attempts which relied largely on deformable component models (DCMs). R-CNN can be broken down into three basic measures. First, it uses the method for selective search which performs the scanning operation for a given input image for necessary objects, producing, on average, 2000 area proposals. Second, utilizing a fine-tuned CNN model, each area proposal’s deep features are extracted and resized to a fixed scale (e.g., 224 × 224). In the final step, to label objects for each region proposal, each region proposal’s features are fed into a set of class-specific SVMs, and the object localizations are optimized using a linear regressor.

### 3.4. ResNet

Human-level image recognition has been achieved using deep CNNs. Low, middle, and high-level features and classifiers are extracted using deep CNNs. Moreover, in-creasing the number of stacked layers would increase the “levels” of features. Microsoft created a platform for deep residual learning, named ResNet [50]. Instead of assuming that a few stacked layers fit a preferred method of mapping directly, they let these layers match a residual mapping. To express F(x) + x, shortcut links with feedforward neural networks are used, specifically links which are of shortcut that bypass one or more layers. Specifically, in-creased depth allows ResNets to achieve greater precision, resulting in better performance than previous networks. To fit dimensions, a stride of two is used in case the shortcuts go through feature maps of two sizes. They are either two layers deep, i.e., ResNet-18 and -34, or three layers deep, i.e., ResNet-50, 101, and 152.

### 3.5. U-Net

The U-Net model is a fast and precise CNN architecture for image segmentation [51]. Convolutional layers were used to construct the model architecture and pixel-based image segmentation. U-Net outperforms traditional models. It also works with images from a minimal dataset. The presentation of this architecture started with the study of biomedical images. The pooling layer, i.e., the method of reducing dimension in height and width that we use in the convolutional neural network, is well-known. By maintaining a constant number of channels in the input matrix, the pooling layer reduces height and width information. In summary, a pixel that reflects groups of pixels is referred to as the pooling layer. The aim of these layers is to improve the output resolution. In the model, the sampled output is combined with high-resolution features for localization. Based on this information, the goal of a sequential convolution layer is to generate more precise results. A segmented output map is generated from the input images. There is no completely linked number of layers in the network model. Only convolution layers are included.

### 3.6. Theoretical Overview of YOLO Family Algorithms

Deep learning algorithms are classified into two categories, (i) one-stage classifiers and (ii) two-stage classifiers. Two-stage classifiers are responsible for generating a region that may include items. These are region are then classified into items by the neural network. Two-stage classifiers generate more results than single-stage classifiers; due to the involvement of the different stages used in the process of detection, they have a slower inference speed. In single-stage detectors, on the other hand, the region defining phase is skipped, and both object classification and detection are completed in one stage. When compared to two-stage classifiers, single-stage classifiers are faster.

YOLO (You Only Look Once) is a category of one-stage classifier deep learning framework that detects objects using a convolutional neural network. It is adopted by many researchers because of its detection speed and accuracy in the result. A variety of deep learning algorithms exist, but none of them can perform object detection in a single run. On the other side, YOLO detects an object in a single run across a neural network, making it ideal to use in real-time applications. Because of these qualities, the YOLO algo-rithm is a frequent choice from among the other algorithms of deep learning framework.

In YOLOv1 image is divided into equal-sized S × S grid cells. If the centre of the items falls inside the cell, then object detection take place on each grid cell. With a confidence score, each cell may anticipate a bounding box with a fixed B number. 5 values such as x, y, w, h, and confidence scores make up each bounding box [52,53]. The centres, width, and height of the bounding box are represented by respectively x, y, w, and h. After predicting a bounding box, IOU (intersection over union) is used by YOLO to find the correct bounding box of an object for the grid cell. Non-max suppression is used by YOLO to reduce extra bounding boxes. If the IOU >= 0.5, then the extra bounding boxes with low confidence score are removed by non-max suppression. The sum of squared error is used by the YOLO to determine loss.

Convolution layers are combined with the batch normalization in YOLOv2 to increase accuracy and minimize the problem of overfitting. To address this problem, in YOLOv3, Darknet 53 replaced the Darknet19 [54] due to its difficulty in detecting small objects. In this paper, the residual block has been incorporated, which skips connections, and performs up-sampling, considerably improving the algorithm’s accuracy. In YOLOv4, Dark-net 53 was updated to CSPDarknet53 to use as the feature extractors’ backbone, which significantly enhanced the algorithm’s speed and accuracy. YOLOv5 is the most recent version of all the YOLO family algorithms, where Darknet was replaced by PyTorch.

**Figure 4:**
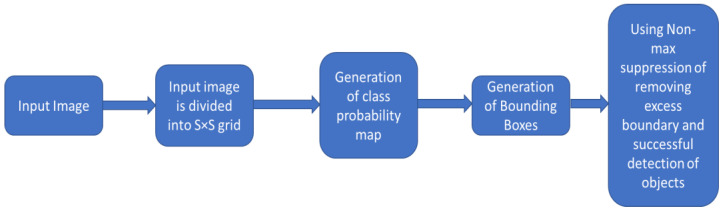
Overall design of YOLO algorithm.

Figure 4 depicts the YOLO algorithm’s overall design, while Table 4 highlights the differences between the YOLO family algorithms’ (YOLOv3, YOLOv4, and YOLOv5 algo-rithm) architectures. All of the algorithms have the same head and neural network type, but different backbone, neck, and loss functions.

Darknet53, the backbone of YOLOv3, extracts feature from an input image. The convolution layer is the backbone of a deep neural network, and its duty is to extract the required information from the input image. As a neck, the feature pyramid network (FPN) is utilized [51]. The neck, which is composed of several bottom-up and top-down paths, is necessary for extracting feature maps from various stages, whereas the head is composed of the YOLO layer. The head’s job is to perform the final prediction, which is a vector of bounding box coordinates: class probability, width, class label, and height, in one-stage detector. Feature extraction is done by supplying the image first to Darknet53, then providing the result generated from the Darknet53 to a FPN. Finally, the YOLO layer is responsible for generating the results.

#### 3.6.1. YOLOv4 Architecture

The cross stage partial network (CSPNet) creates a new feature extractor backbone called CSPDarknet53 in the YOLOv4 Darknet, which is a modified version of YOLOv3. DenseNet [55,56], which has been upgraded, provides the basis for the convolution design. Dense block is used to transfer a copy of the feature map from the base layer to the next layer. DenseNet offers a lot of benefits, including lesser gradient vanishing difficulties, improved learning, improved backpropagation, and the removal of the processing bottleneck. The neck is formed using the spatial pyramid pooling (SPP) layer and the path aggregation network (PANet). The SPP layer and PANet are used to increase the receptive field and to extract important data from the backbone. A YOLO layer is also present on the head. Feature extraction from the image is performed by giving the image to CSPDarknet53, then supplied to PANet for feature fusion. Finally, identical to YOLOv3, the results are generated by the YOLO layer. YOLOv4 algorithm performance can be improved by utilizing the bag of freebies [57] and bag of specials [57]. Different techniques of augmentation, drop block regularization, and complete IOU loss (CIOU), are included in the bag of freebies. Diou-NMS [58] modified the path aggregation network, and mish activation is included in the bag of specials.

#### 3.6.2. YOLOV5 Architecture

**Figure 5:**
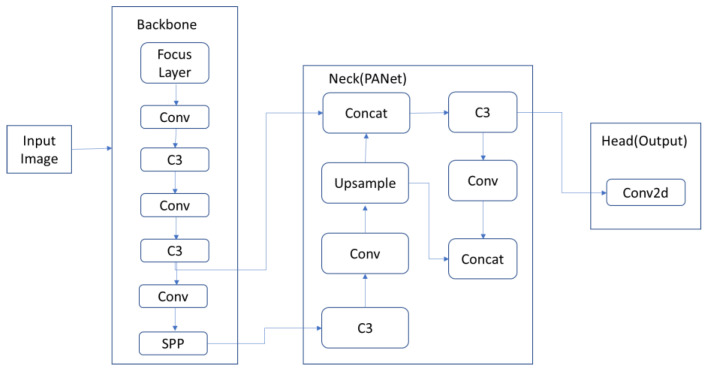
Architecture of YOLOv5 algorithm.

YOLOv5 is more like its prior version release. PyTorch is used instead of Darknet. CSPDarknet53 is used as a backbone. The repeating gradient information in big backbones and integration of gradient change into the feature map are handled by the backbone, which speeds up inference, improves the accuracy parameter, and decreases the size of the model by lowering parameters. To boost information flow, a path aggregation network (PANet) is used as a neck. A new FPN with many bottom-up and top-down layers is used by PANet. This enhances the model’s low-level feature propagation. PANet improves the object’s localization accuracy by improving localization in lower levels. Moreover, the YOLOv5 head is identical to that of YOLOv4 and YOLOv3, resulting in three distinct feature map outputs for multiscale prediction. It also contributes towards the efficient prediction of small to large objects in the model. Feature extraction from the image is performed by giving the image to CSPDarknet53. Then, for feature fusion, PANet is used. Finally, the results are generated by the YOLO layer. The YOLOv5 algorithm’s architecture is shown in Figure 5 The Focus layer evolved from the YOLOv3 framework [59].

In YOLOv5, the first three layers of YOLOv3 are replaced by the single Focus layer. Furthermore, there is a convolution layer denoted by Conv. There are three convolution layers denoted by C3 and a module that is connected by bottlenecks. The network’s fixed size constraint is removed by the SPP (spatial pyramid pooling) pooling layer, upsam-pling the previous layer fusion in the adjacent node. Concat is a slicing layer that slices the layer before it. The last three Conv2d modules are detection modules that are employed in the network’s head.

The architectures of the three YOLO family algorithms, namely YOLOv3 algorithm, YOLOv4 algorithm, and YOLOv5 algorithm, are fundamentally different. YOLOv3 leverages the Darknet53 backbone, CSPdarknet53 is used in the YOLOv4 design, while the Focus layer is used in the YOLOv5 architecture. The Focus layer is originally introduced in the YOLOv5 algorithm. The Focus layer replaces the first three layers in the YOLOv3 algorithm. A Focus layer has the advantage of requiring less CUDA memory, having fewer layers, and allowing for more forward and backward propagations [59].

## 4. Experimental Results

### 4.1. Experimental Setup

The methodology proposed in the paper was tested and executed on a personal computer with the specifications as Intel(R) Core (TM) i5-8250U processor (Intel Corporation, Santa Clara, CA, USA), NVIDIA^®^ TITAN XP^®^ 3840NVIDIA CUDA^®^ architecture with 12 GB GDDR5X RAM of 16 GB (NVIDIA Corporation, Santa Clara, CA, USA), storage of 1TB SSD, and Microsoft Windows 10 operating system (Microsoft, Redmond, WA, USA).

To train the neural networks, the open-source google colab has been used along with Tesla P100-PCIE-16GB graphics cards (NVIDIA Corporation, Santa Clara, CA, USA). Different types of Google cloud computing services are offered, such as paid and free, which can be utilized in a variety of computer applications. In terms of accuracy, it can be said that YOLOV5I outperforms YOLOv4 and YOLOV3. We used the PyTorch framework to train YOLOv5, and as the YOLOv4 algorithm and YOLOv3 algorithm are built in the Darknet framework, the Darknet framework is used to train YOLOv3 and YOLOv4.

### 4.2. Evaluation Metrics

The F1 score and the mAP are used to compare the performance of the three YOLO family algorithms, namely YOLOv3, YOLOv4, and YOLOv5. As seen in Equation (3), the F1-score can be defined as the harmonic mean value of precision and recall [60]. It’s also the model’s test accuracy. The highest possible F1 score value is 1, which indicates perfect precision and memory, while the lowest possible value is 0, which indicates either zero precision or recall. Furthermore, an average of the average precision of all the classes can be defined as a mAP, indicated in Equation (5), where the number of queries is denoted by q and average precision for that query is denoted by AveP(q). The mean of AP can then be used to determine mAP. To determine the accuracy of machine learning algorithm a mAP metric is also used. The true positive is the total number of ships detected by the algorithm from satellite images. The number of false positives is the number of non-ship objects that the algorithm mistakenly detects as a ship, and the number of false negatives is the number of ships that the algorithm fails to detect. Furthermore, the algorithms’ inference speed can be determined by a frame per second (FPS). The frame rate is inversely proportional to the time it takes to process a single frame of video. IOU is the ratio of the ground truth label’s area of overlap to the prediction label’s area of union. To determine if a prediction is true positive or false positive, an IOU threshold value was utilized. The average precision is then calculated using the precision-recall curve. A mAP is defined as the average of the average precision of all the classes, as previously indicated. It’s worth mentioning that accuracy is defined as the percentage of true predictions to total predictions. A model’s precision is 100 percent if 50 predictions are produced and all of them are correct. Precision is defined as the number of true objects in an image that are not considered, as shown in Equation (1). On the other hand, recall is defined as the ratio of correct predictions to the overall number of objects in the image, as shown in Equation (2). For instance, if a model predicts 65 true objects in an image with 100 genuine objects, the recall is calculated to be 65%. One cannot imply that the model is accurate if it shows high precision and recall. An algorithm is stated as accurate if it has a good balance between precision and recall. To determine this, the same F1- score is used to check whether the model is accurate or not.

The paper aims to develop a real-time algorithm that can be utilized on a Personal Computer (PC), specifically for detecting ships from satellite images which are small in size.

Precision = TP/(TP + FP)(1)

Recall = TP/(TP + FN)(2)

F-Measure = 2 (Precision · Recall)/(Precision + Recall)(3)

Accuracy = (TP + TN)/(TP + TN + FP + FN)(4)

mAP = ∑^Q^_q = 1_ AveP (q)/Q (5)

### 4.3. Analysis of the Results

To train the neural network, we first utilized YOLOv3 as a training optimizer using stochastic gradient descent and 0.9 set as the momentum. Respectively, 0.001 and 0.0005 were the learning rate and weight decay. The training images’ height and width were 416 × 416 pixels, respectively.

The YOLOv4 and YOLOv5 have been trained the same way the YOLOv3 algorithm was trained, i.e., with identical parameters. Table 5 compares the three YOLO algorithms for the detection of ships from the Airbus Ship Challenge and Shipsnet satellite image dataset. When compared to YOLOv3 and YOLOv4, YOLOv5 has a better mAP and F1 score, indicating that YOLOv5I shows greater accuracy in the detection of objects than YOLOv3 and YOLOv4 for the Airbus Ship Challenge and Shipsnet satellite images dataset. In this investigation, the YOLOv3 was found to be faster than the YOLOv4 and YOLOv5. Because it employs auto learning bounding boxes [61,62], YOLOv5 has a higher accuracy than YOLOv4, which improves the algorithm’s accuracy. Because YOLOv3 uses Darknet53, which has problems recognizing a tiny item, and YOLOv4 and YOLOv5 employ CSPdarkent53, which increases overall accuracy, YOLOv4 and YOLOv5 show higher accuracy than YOLOv3. YOLOv5

**Figure 6:**
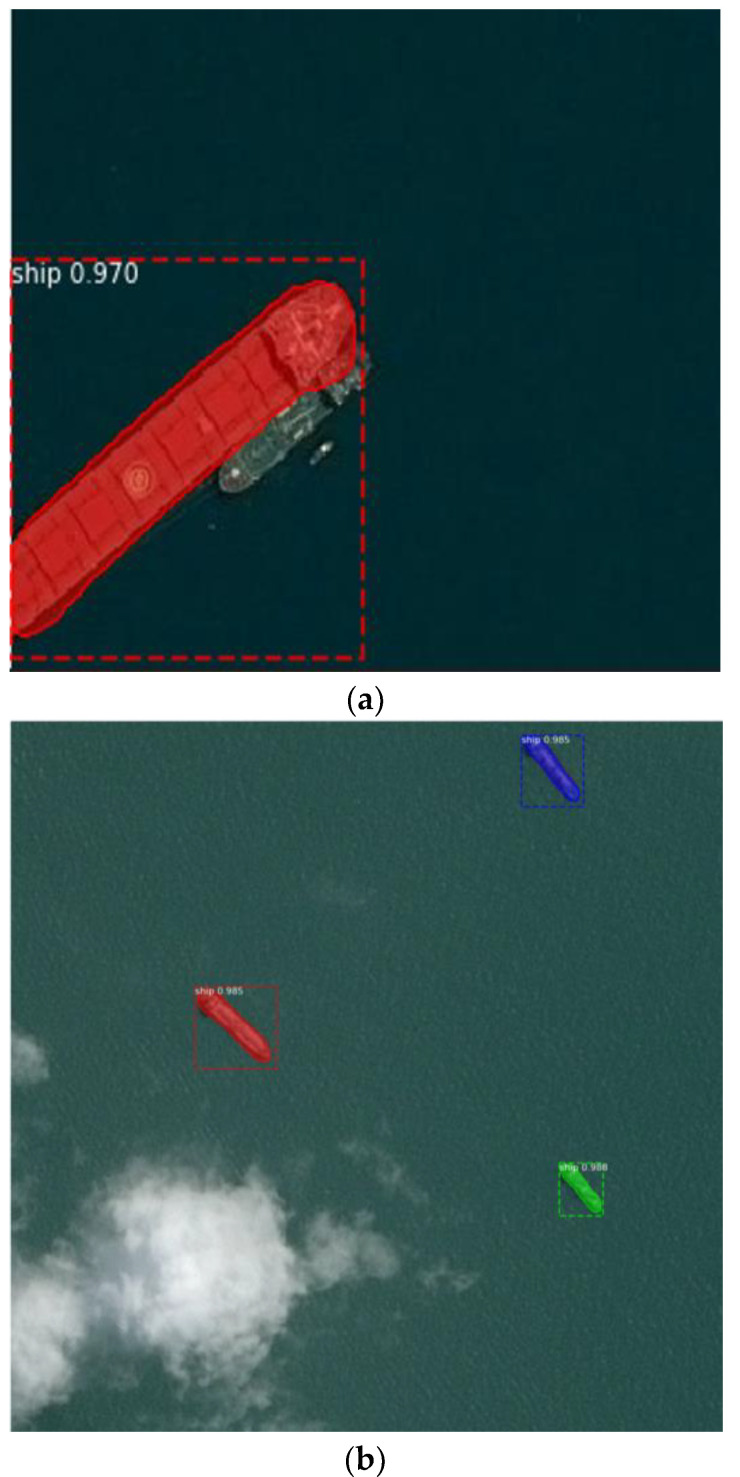

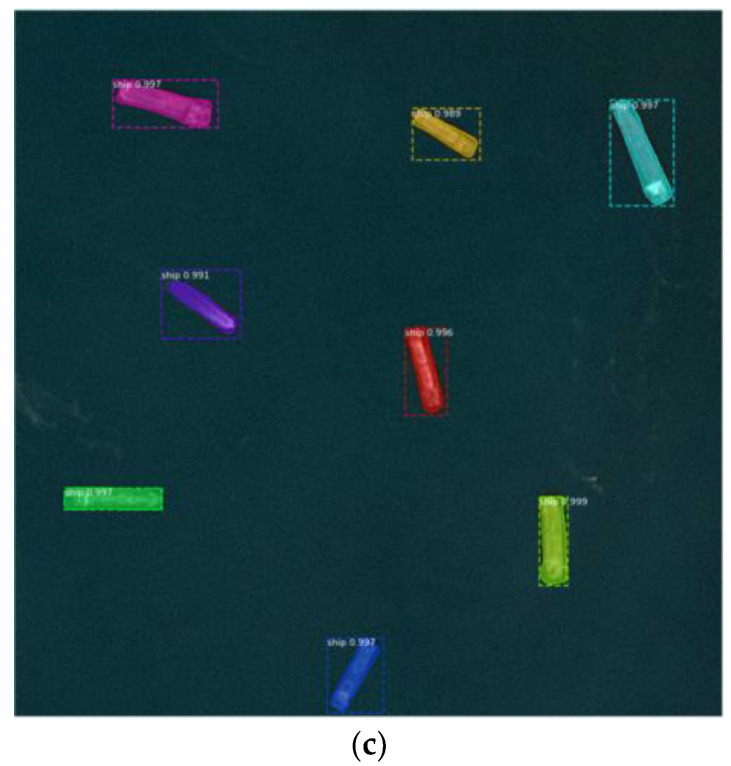
Result of Ship Detection using YOLO family algorithms: (**a**) YOLOv3 (**b**) YOLOv4 (**c**) YOLOv5.

**Figure 7:**
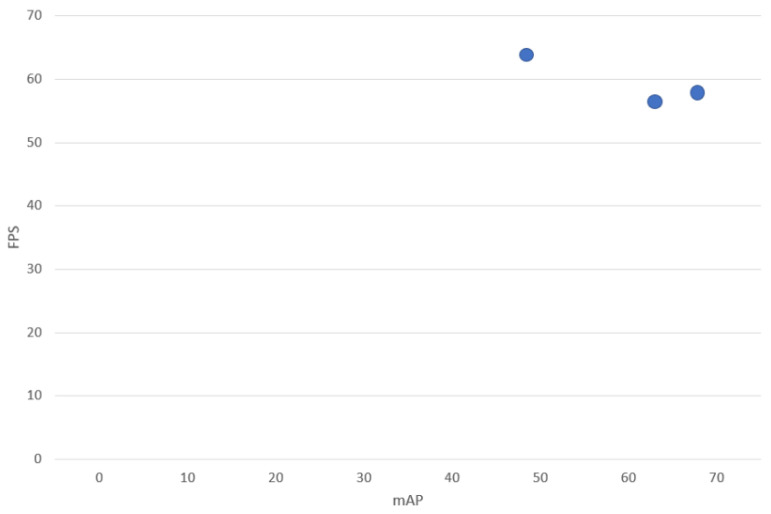
Performance of YOLOv3, YOLOv4, and YOLOv5 on PC.

The result of the application of the YOLO family algorithms to a satellite image is shown in Figure 6. In addition, Figure 7 depicts the performance of YOLO algorithms on a PC.

Table 5 displays the algorithms’ accuracy and recall. Table 6 provides the three YOLO algorithms, average precision results of the ship labels. YOLOv3 shows a high precision value but a low recall value, indicating that the YOLOv3 model has to be improved. For its use, there has to be a balance between precision and recall, which in turn is again reflected in the method’s F1 score. Precision and recall are balanced in YOLOv4 and YOLOv5, as can be seen. As a result, YOLOv4 and YOLOv5 have greater F1 scores than YOLOv3, despite the fact that YOLOv3 shows high precision. It has been observed that a good balance of precision and recall in YOLOv4 and YOLOv5 has resulted in a high F1 score.

Figure 7 show that all three algorithms render good results in ship detection. Accuracy is a key criterion to consider when selecting the best algorithm for detecting ships. According to the result received in Table 5 and Figure 7, we can conclude that YOLOv5I is best as it has the highest accuracy in detecting ships from satellite images. Moreover, keep in mind that these algorithms have been evaluated on a PC to verify the results. On a PC, YOLOv5 achieves greater accuracy than other YOLO family algorithms, such as YOLOv3 and YOLOv4. Employing YOLOv5 resulted in a modest speed loss (2.5 FPS) in comparison to the YOLOv3 algorithm.

## 5. Conclusions and Future Work

The main focus of this paper has been to evaluate an object detection system which detects ships from satellite images. YOLOv3 and YOLOv4 were tested, which are well-known in the scientific community, and YOLOv5 was used in an experimental ship detection task, comparing YOLOv3 and YOLOv4 architectures to demonstrate that YOLOv5I outperforms them in terms of detection accuracy of 99% compared to 98% and 97% respectively for YOLOv4 and YOLOv3. The algorithms’ performance was compared to see which method gave the best results in ship detection. For training, testing, and validation, the Airbus Ship Challenge and Shipsnet satellite datasets were used, and the YOLO family algorithms were then evaluated on a PC. According to the findings of our experiments, all three algorithms satisfy the criteria for the ship detection. Given the results, YOLOv5 has been chosen as the method with the highest accuracy in the detection of ships from satellite images. Thus, integrating YOLOv5 with the Airbus Ship Challenge and Shipsnet satellite datasets for ship detection can be done quickly and accurately.

Future lines of research are going to focus on the incorporation of location features into the object detection/classification scheme in order to determine the location of the ship. Moreover, new experiments must be carried out to test the different sensors, such as SARs, under extreme conditions, where visible spectrum imagery is unavailable, e.g., at night, when it is cloudy, or if there is fog. Additionally, different sensors can be integrated in a multi-modal scenario and saliency estimation methods may be used to not only classify whether or not an image includes a ship, but also to obtain the exact position of the ship being identified and the other objects, such as yachts, boats, and aircraft. Future work will also entail the adaption and usage of the range of available HPC (high performance computing) resources.

## Figures and Tables

**Table 1:** Comparative Analysis of various Research work on Object Detection.

Paper Referred	Dataset	Pixel Size and Resolution	Framework/Algorithm	Precision/Recall/Accuracy
[23]	SPOT-5 and Google Earth Services	Pixel Size 9000 × 9000 and Resolution 5 m	Proposed method-based sea surface analysis	Precision- 89.22% & Recall- 97.80%
[30]	ImageNet LSVRC-2010		deep convolutional neural network	Precision- 78.1% & Recall- 60.9%
[32]	high-resolution remote sensing (HRRS)	Pixel Size 600 × 600	CNN	Accuracy- 94.6%
[35]	Kaggle Dataset	Pixel Size 768 × 768	UNet	Accuracy- 82.3%
[36]	Airbus Satellite Image Dataset	Pixel Size 768 × 768	CNN based Deep Learning	Accuracy- 89.7%
[37]	RADARSET-2 and Sentinel-1		Faster R-CNN	Precision- 89.23% & Recall- 89.14%
[38]	SAR Ship Detection Dataset (SSDD)		Knowledge Transfer Network and CNN based detection model	Precision- 98.87% & Recall- 90.67%
[39]	WorldView-2 and -3, GeoEye and Pleiades	Resolution between 0.3 m and 0.5 m	YOLOV2, YOLOV3, D-YOLO and YOLT	Average Precision- 60% for vehicle and 66% for vessel
[40]	Google Earth Images	Resolution be-tween 2 m and 0.4 m	Two staged CNN-based ship detection technique	Accuracy- 88.3%
[41]	Google Earth Images	Pixel Size ranges from 900 × 900 to 3600 × 5400	Transfer learned Single-shot Multibox Detector (SSD)	Accuracy- 87.9%

**Table 2:** Class-wise total number of sample images.

Class	Number of Images
Non-ship	3000
Ship	1000

**Table 3:** Class-wise images of ship.

Class	Image of Ship
Non-ship	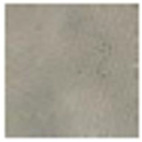
Ship	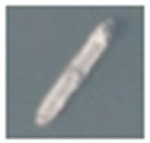

**Table 4:** The Architecture differences between the YOLO family algorithms.

Parameters	YOLOv3 Algorithm	YOLOv4 Algorithm	YOLOv5 Algorithm
Type of Neural Network	Fully Convolutional	Fully Convolutional	Fully Convolutional
Feature Extractor	Darknet-53	CSPDarknet53	CSPDarknet53
Neck	FPN	SSP and PANet	PANet
Head	YOLO layer	YOLO layer	YOLO layer

**Table 5:** Ship Detection results’ comparison using YOLOv3, YOLOv4 and YOLOv5 algorithms.

Evaluation Measures	YOLOv3 Algorithm	YOLOv4 Algorithm	YOLOv5 Algorithm
F1-Score	0.56	0.58	0.61
Recall	0.44	0.59	0.63
Precision	0.71	0.67	0.70
mAP	0.49	0.61	0.65

**Table 6:** Ship Detection-Average Precision Performance of YOLOv3, YOLOv4 and YOLOv5 algorithm.

Label	YOLOv3	YOLOv4	YOLOv5
Ship	73.27	84.19	80.7

## Data Availability

Data available in a publicly accessible repository that does not issue DOIs. Publicly available datasets were analyzed in this study. This data can be found here: [https://www.kaggle.com/c/airbus-ship-detection/data] (accessed on 11 August 2021) and [https://www.kaggle.com/rhammell/ships-in-satellite-imagery] (accessed on 28 October 2021).
